# The impact of night shift workload on nurses’ depressive symptoms: a chain mediation analysis of sleep disturbances, social avoidance and fear of missing out

**DOI:** 10.3389/fpubh.2025.1723567

**Published:** 2025-12-17

**Authors:** Meiting Jiang, Wenfei Yang, Yiyang Ji, Xin Cai, Yiye Li, Aijun Guan, Zhizhi Wang, Caihua Ye, Shaoying Tan, Xiating Li, Dongyang Zeng, Xiang Zhang

**Affiliations:** 1 Nursing Department, The First Affiliated Hospital of Hainan Medical University, Haikou, China; 2 School of Nursing, Hainan Medical University, Haikou, China; 3 Nursing Department, Haikou People's Hospital, Haikou, China

**Keywords:** depressive symptoms, fear of missing out, night shift work, nurse, sleep disturbances, social avoidance and distress

## Abstract

**Background:**

Depression is highly prevalent among nursing staff and has become a globally recognized occupational health issue. Despite its significance, research exploring the complex mechanisms linking night shift work and depressive symptoms in nurses remains limited.

**Objective:**

This study aims to investigate the association of sleep disturbances, social avoidance and distress (SAD), and fear of missing out (FOMO) with night shift work and depressive symptoms among nurses.

**Methods:**

Between June and August 2025, clinical nurses from a Grade-A tertiary hospital in Haikou were recruited. Data were collected using a general-information questionnaire, the 10-item Center for Epidemiologic Studies Depression Scale, the Pittsburgh Sleep Quality Index Scale, the Social Avoidance and Distress Scale, and the Fear of Missing Out Scale. Analyses were performed with SPSS 25.0 and R Studio 4.4.1. Group comparisons were conducted using the Wilcoxon rank-sum or Kruskal-Wallis *H* tests. Spearman correlation analyses examined associations among variables. Mediation analysis was performed with the PROCESS 4.0 program. The significance of indirect effects was tested by bootstrapping and structural equation modelling.

**Results:**

A total of 614 clinical nurses were ultimately enrolled, of whom 36 (5.9%) were male and 578 (94.1%) were female. The mean depression score was 9.38 ± 5.42, corresponding to a positive screening rate of 50.81%. Mediation analyses showed that night shift workload positively predicted depressive symptoms (*β* = 0.0416, 95% *CI*: 0.0351–0.0481). Sleep disturbance, SAD, and FOMO each emerged as significant partial mediators. The indirect effects were 0.0381 (95% *CI*: 0.0307–0.0459), 0.0120 (95% *CI*: 0.0078–0.0168), and 0.0122 (95% *CI*: 0.0080–0.0170), accounting for 29, 9.13, and 9.28% of the total effect, respectively. The structural equation model indicated a good fit for the proposed mediation framework.

**Conclusion:**

Night shift workload positively predicts depressive symptoms among nurses, and sleep disturbance, SAD, and FOMO partially mediate this relationship. Comprehensive, multi-target interventions should therefore be implemented to lower depression risk and safeguard the mental health of nurses working night shifts.

## 1 Introduction

Nurses constitute an indispensable core of global healthcare systems, representing the largest professional group among frontline healthcare workers and accounting for approximately 59% of all healthcare professionals (1). They play a vital and irreplaceable role in assisting diagnosis and treatment, saving lives, alleviating suffering, and promoting patient recovery (2). However, with societal development and rising healthcare demands, the expectations placed on the nursing profession have significantly increased (3). Nursing responsibilities extend beyond responding to public health emergencies and providing disease-related care; they also include managing patients’ daily activities during hospitalization and ensuring medication safety, which has substantially intensified workload demands (4). Factors such as high-intensity work, inadequate staffing, occupational exposure risks, and strained nurse–patient relationships make nurses particularly vulnerable to mental health challenges, notably anxiety and depression (5). Depression in nurses typically manifests through loss of interest in daily activities, persistent low mood, sleep disturbances, appetite changes, and reduced energy levels (6). Recent statistics reveal that the prevalence of depression among nurses in China is as high as 43.83% (7). Such depressive symptoms not only undermine nurses’ physical and mental health and overall quality of life but also negatively affect nursing service quality and patient satisfaction, while increasing the risk of medical disputes (8). Consequently, early identification of risk factors and timely interventions targeting depression among Chinese nurses are essential for safeguarding their wellbeing and improving the quality of nursing care.

Night shift work represents an indispensable component of the nursing profession. Due to the continuous and unpredictable nature of healthcare services, nurses, as frontline clinical caregivers, are often required to work night shifts (typically from 22:00 or 23:00 until 07:00 or 08:00 the following day). During these hours, they are responsible for monitoring vital signs, carrying out medical orders, responding to clinical changes, and ensuring patient safety, thereby maintaining the continuity and security of healthcare delivery (9). However, night shifts are also recognized as a unique occupational stressor, associated not only with metabolic disorders, cardiovascular disease, diabetes, and breast cancer, but also with a substantially increased risk of depression (10). Extended working hours and demanding tasks at night readily contribute to psychological stress and fatigue, further compounding nurses’ physical and mental strain (11). Thus, while night shifts safeguard uninterrupted healthcare, urgent attention must be directed toward the mental wellbeing of night-shift nurses.

Existing research further demonstrates that prolonged and frequent night shifts disrupt sleep cycles, leading to sleep disorders, a critical risk factor for depressive symptoms (12). Night work requires nurses to function outside the natural sleep–wake rhythm. Since the human body’s biological clock naturally favors nocturnal rest and daytime activity, night shift work creates a significant conflict with this natural rhythm, easily triggering a series of sleep problems such as disrupted sleep patterns and reduced sleep quality. Common manifestations include difficulty falling asleep, early awakening, and decreased total sleep duration (12). Nursing practice, however, demands sustained concentration and vigilance to ensure patient safety and the provision of high-quality care. In such high-pressure environments, sleep disturbances often worsen (13). Studies have shown that chronic sleep disorders not only impair nurses’ daily functioning and quality of life but also disrupt emotional regulation, thereby increasing vulnerability to depressive symptoms (14).

Social avoidance and distress (SAD) is a psychological condition characterized by avoidance behaviors in social contexts and negative emotional responses. It primarily manifests as reduced participation in social and group activities due to anxiety, tension, or embarrassment in anticipation of social interactions (15). SAD extends beyond actual social encounters to include aversion toward social situations more broadly (16). Prolonged SAD can hinder the formation and maintenance of interpersonal relationships, negatively impacting work performance, quality of life, and serving as significant risk factors for depression and anxiety (17). Additionally, nurses often experience exhaustion from external interactions following night shifts, which reduces their willingness to engage and leads to avoidance of social activities. Over time, such social isolation can foster feelings of loneliness and low mood (18).

Fear of missing out (FOMO), also referred to as the anxiety of missing out, describes an individual’s apprehension when faced with the possibility of missing significant or enjoyable events (19). Research shows that FOMO occurs across all age groups and is closely associated with self-esteem, sense of belonging, and social recognition (20). Statistics indicate that approximately 78.3% of young people engage persistently in various social activities due to FOMO, with around 15.2% exhibiting severe symptoms (21). FOMO often leads individuals to frequently check and refresh social media feeds, monitoring others’ ongoing activities and experiences while feeling disappointed and anxious about their own non-participation (22). Nurses working night shifts experience misaligned schedules relative to conventional social activity times, frequently missing opportunities to gather with family or friends or participate in leisure pursuits (23). This effect is further amplified by social media, where exposure to others’ shared updates can intensify anxiety and feelings of loss regarding missed social opportunities (24). Consequently, FOMO may contribute to emotional isolation and a diminished sense of belonging, acting as a significant precursor to depressive symptoms (25). According to the dispositional-stress model of depression, individuals with inherent psychological vulnerabilities, such as high FOMO tendencies, are more susceptible to depression when encountering external stressors like social isolation or missed experiences (26).

The stress-process model proposes that external, structural stressors impair emotional health and social adaptation by triggering secondary strains and psychological mediators (27). Night-shift work is considered a prototypical primary stressor whose impact accumulates through a chain of internal transmissions (28). When nurses are chronically exposed to circadian-disrupting schedules, biological rhythm disturbance first leads to sleep disturbance. This disturbance not only undermines emotion regulation but also depletes cognitive and affective resources necessary for interpersonal engagement (29). Because their off-duty hours are out of phase with conventional social rhythms, nurses experience difficulty integrating into everyday interaction cycles. This situation increases interpersonal avoidance, tension, and sensitivity to negative evaluation, factors that precede social anxiety (30). When heightened social anxiety combines with schedules markedly misaligned with mainstream society, real-world social contact decreases, and individuals often turn to social media for a sense of belonging. Hyper-vigilance towards others’ online activity and fear of being excluded from group interactions lead to information overload, social comparison, and an intensified sense of “missing out,” thereby increasing FOMO (31). Persistent FOMO, in turn, amplifies loneliness, self-worth doubts, and emotional exhaustion, leading directly to depressive symptoms (21). Thus, the stress process is progressively amplified: if sleep disturbance triggered by night-shift work is not effectively addressed, social anxiety and FOMO may escalate, ultimately damaging emotional health and manifesting as depressive symptoms.

Although previous research has demonstrated a significant association between night-shift work and depressive symptoms (32), the roles of sleep disturbance, SAD, and FOMO in this relationship remain unexplored. Therefore, this study investigated how sleep disturbance, SAD, and FOMO mediate the relationship between night-shift workload and depressive symptoms among nurses. The findings aim to provide new evidence to support effective psychological interventions and promote improved mental health in this population.

Based on this rationale, the following hypotheses were proposed: H1. Night-shift workload positively predicts depressive symptoms in nurses. H2. Sleep disturbance mediates the relationship between night-shift work and depressive symptoms. H3. SAD serves as an important mediator between night-shift workload and depressive symptoms. H4. FOMO is also related to the association between night-shift workload and depressive symptoms.

## 2 Methods

### 2.1 Study design and participant recruitment

This study employed a cross-sectional research design to examine clinical registered nurses at a public hospital in Hainan Province, China. Convenience sampling was used to recruit participants from among the hospital’s clinical registered nurses. Inclusion criteria required participants to hold a valid Chinese Registered Nurse practicing certificate and to provide voluntary informed consent. Exclusion criteria were applied to non-permanent hospital staff, including trainee nurses and student nurses.

In this study, the minimum sample size was calculated using the single-proportion formula: *n* = *Z*^2^*α*/2 × *p*(1−*p*)/*d*^2^ (8). The parameters were set as follows: the prevalence of depressive symptoms among nurses in China was 43.83% (7), the margin of error was *d* = 5%, and the confidence level was 95% (*α* = 0.05, corresponding to *Zα*/2 = 1.96). Substituting these values into the formula yielded *n* = (1.96)^2^ × 0.4383 × (1–0.4383)/0.05^2^ = 378.32, which was rounded up to 379. To account for potential non-response or incomplete data, a 20% sample size buffer was applied, resulting in 379/(1–0.2) = 473.75. Therefore, the final required minimum sample size was determined to be 474 nurses.

### 2.2 Measurement

#### 2.2.1 Depressive symptoms

The Centre for Epidemiologic Studies Depression scale (CESD-10) was used to assess depressive symptoms among nurses. This abbreviated version evaluates depressive symptoms experienced over the preceding week, focusing on emotional experiences and behavioral manifestations. The scale employs a four-point rating system: “rarely or almost never” scores 0 points, “not too much (1–2 days)” scores 1 point, “sometimes or about half the time (3–4 days)” scores 2 points, and “most of the time” scores 3 points. Total scores range from 0 to 30, with higher scores indicating more severe depressive symptoms. The CESD-10 has been validated in Chinese populations and widely used in Chinese epidemiological and occupational health studies, demonstrating good psychometric properties (33). In the present study, Cronbach’s *α* was 0.902, indicating excellent internal consistency.

#### 2.2.2 Night shift workload

In this study, night shift workload intensity was measured using a night shift intensity coefficient, calculated as follows: Night Shift Intensity Coefficient = Monthly Night Shift Frequency × Night Shift Duration × Department Tier Coefficient. A higher coefficient indicates a greater night shift workload for nurses. The monthly frequency of night shifts refers to the actual number of night shifts a nurse works each month, starting from the previous evening or afternoon and continuing until the following morning. Night shift duration represents the total working hours from the nurse’s arrival at the workplace until their departure the next day. The department tier coefficient is based on the performance tier classification of the units surveyed: Tier 1 includes the Emergency Department and all Intensive Care Units; Tier 2 comprises Operating Theatres and Delivery Rooms; and all other departments are classified as Tier 3.

#### 2.2.3 Sleep disorder

The PSQI is a standardized instrument developed by psychiatrist Buysse and colleagues at the University of Pittsburgh in 1989 to assess perceived sleep quality (34). In 1996, Liu et al. introduced the scale to China and conducted psychometric testing, supporting its reliability and validity in Chinese populations (35). The self-report questionnaire evaluates sleep quality over the previous month through 18 items grouped into seven components: subjective sleep quality, sleep latency, sleep duration, habitual sleep efficiency, sleep disturbances, use of sleeping medication, and daytime dysfunction. Each component has scores ranging from 0 to 3, resulting in a global score of 0–21. Higher scores indicate worse sleep quality (36). In the present study, the internal consistency of the PSQI was good (Cronbach’s *α* = 0.834).

#### 2.2.4 Social avoidance and distress

The Social Avoidance and Distress Scale (SADS), developed by Watson et al., was initially designed to assess individuals’ social avoidance behaviors and socially related distress. A Chinese version was developed by Wang et al. (37) in 1993 and has since been widely used and validated in China. Test–retest reliability is 0.68, and the scale demonstrates satisfactory internal consistency and validity. It contains two 14-item subscales—social avoidance and social distress—giving 28 items in total. Each item is answered “yes” (1) or “no” (0), so total scores range from 0 to 28, with higher scores indicating greater avoidance and distress. The SADS is one of the most frequently employed standardized measures of social avoidance and distress and has been applied to university students, older adults, cancer patients, and other Chinese samples (16). In the present study, the scale showed good internal consistency (Cronbach’s *α* = 0.872).

#### 2.2.5 Fear of Missing Out

The Chinese version of the Fear of Missing Out Scale (FOMO) was developed by Li in 2019 through translation and adaptation of the original scale. This questionnaire consists of two dimensions: Fear of Missing Information and Fear of Missing Situations, each containing four items, for a total of eight items. It uses a five-point Likert scale ranging from “1 = Not at all true” to “5 = Completely true,” with higher total scores indicating a stronger FOMO. The scale has demonstrated validity in Chinese populations and is suitable for assessing FOMO among Chinese respondents (38). In this study, the scale’s internal consistency was good (Cronbach’s *α* = 0.764).

#### 2.2.6 Control variables

A general information questionnaire was developed based on previous research to collect basic information from nurses, primarily encompassing demographic characteristics and work-related attributes. Demographic variables included gender (male, female), age (20 ≤ years<30, 30 ≤ years<40, years ≥ 40), marital status (married, unmarried/divorced/separated), and educational attainment (college diploma or below, bachelor’s degree, postgraduate degree). Work-related characteristics included years of service (<5 years, 5–10 years, 11–15 years, >15 years), professional title (Nurse, Senior Nurse, Head Nurse, Deputy Chief Nurse and above), monthly income (<5,000, 5,000–8,000, >8,000), department (internal medicine, surgery, obstetrics and gynecology, pediatrics, emergency department, intensive care unit, outpatient clinics, and other departments), as well as the monthly number and duration of night shifts.

#### 2.2.7 Data collection

The electronic questionnaire was designed and distributed via Wenjuanxing.^1^ A link to the survey was sent to the target nurses through WeChat, a widely used social-media app. To minimize missing data, the questionnaire was programmed to allow submission only after all items had been completed. A total of 634 nurses accessed the survey; after removing 20 low-quality responses, 614 valid questionnaires remained, yielding an effective response rate of 96.8%.

### 2.3 Statistical analysis

Data analysis was conducted using SPSS 25.0 software, R Studio 4.4.1 software, and Hayes’ PROCESS 3.5.2 add-on. Firstly, descriptive statistics summarized participants’ sociodemographic characteristics. Continuous variables did not pass Shapiro–Wilk normality tests and were therefore reported as median and interquartile range [*M* (*IQR*)]. Intergroup comparisons were performed using the Wilcoxon Mann–Whitney *U* test and Kruskal-Wallis *H* test. Categorical variables were presented as frequency and percentage, with intergroup comparisons conducted using chi-square tests. Spearman’s correlation analysis was performed to examine relationships among night-shift work intensity, depression, sleep disturbance, SAD, and FOMO. PROCESS Model 6 was applied, with depression as the dependent variable, night-shift work intensity as the independent variable, and sleep disturbance, SAD, and FOMO as mediating variables. The significance of mediating effects was assessed using the bootstrap method, with 5,000 resamples to calculate bias-corrected 95% confidence intervals. Additionally, structural equation modelling was conducted to evaluate the stability of the mediation model. All statistical tests were two-tailed, with the significance level set at *α* = 0.05.

### 2.4 Ethical approval and consent to participate

This study was approved by the Ethics Review Committee of the First Affiliated Hospital of Hainan Medical University (Approval No.: 2024-KYL-077). All participants provided informed consent prior to participation. All procedures were conducted in accordance with relevant guidelines and regulations.

## 3 Results

### 3.1 Participant characteristics

A total of 614 nurses were included in this survey. The median age was 34 years (range 22–53). Among them, 578 (94.1%) were female and 36 (5.9%) were male; 418 (68.1%) were married and 196 (31.9%) were unmarried. Regarding education, 91.2% held a bachelor’s degree, and 1.3% held a postgraduate degree. Professional titles were classified as junior level (213, 34.7%), intermediate level (381, 62.1%), and senior level (20, 3.3%). Analysis indicated statistically significant differences in depression scores across departments (*p* < 0.001; Table 1). Further multiple comparisons using Dunn’s method with Bonferroni correction (Table 2) showed that nurses in critical care departments had significantly higher depression scores compared to those in outpatient and other departments (*Z* = −4.583, *p* < 0.001) and internal medicine, surgery, obstetrics, and pediatrics (*Z* = −2.906, *p* = 0.004). Although depression scores trended higher compared to emergency/operating theatre nurses, this difference was not statistically significant (*Z* = −1.007, *p* = 0.314).

**Table 1:** The general characteristics of participants (*N* = 614).

Variable	Category	*N* (%)/*M* (*IQR*)	Depressive symptoms, *M* (*IQR*)	*Z/H*	*p*
Gender	Male	36 (5.9)	10 (3.5, 13)	−0.327^a^	0.744
	Female	578 (94.1)	10 (5, 12)		
Age	20 ≤ years < 30	142 (23.1)	10 (6, 13)	4.101	0.129
	30 ≤ years < 40	381 (62.1)	10(5, 12)		
	Years ≥ 40	91 (48)	8 (4, 11)		
Marital status	Married	418 (68.1)	9 (5, 12)	−1.428^a^	0.153
	Single/separation/divorced	196 (31.9)	10 (6, 13)		
Educational status	College or below	46 (7.5)	7 (3, 12)	4.553	0.103
	Undergraduate	560 (91.2)	10 (5, 12)		
	graduate	8 (1.3)	9.5 (3.5, 13)		
Years of experience	<5 years	115 (18.7)	10 (5, 13)	4.199	0.241
	5-10 years	124 (20.2)	10 (5, 12.8)		
	10-15 years	240 (39.1)	10 (5, 13)		
	>15 years	135 (22)	9 (4, 12)		
Professional title	Primary	213 (34.7)	10 (5, 12)	0.154	0.926
	Intermediate	381 (62.1)	10 (5, 12)		
	Senior	20 (3.2)	8.5 (5, 13.5)		
Monthly income	<5,000	65 (10.6)	11 (6, 14.5)	3.323	0.190
	5,000–8,000	311 (50.6)	10(5, 13)		
	>8,000	238 (38.8)	10 (5, 12)		
Department	Internal Medicine, Gynaecology and Paediatrics	482 (78.5)	10 (5, 12)	24.072	<0.001
	Emergency Department	34 (5.5)	11 (5, 14.3)		
	Intensive Care Unit	38 (6.2)	12 (7.75, 14.3)		
	Outpatient and Other Departments	60 (9.8)	5 (3, 11)		

a Denotes *Z*-values; all others are *H*-values. *Z*, Mann–Whitney U test; H, Kruskal-Wallis test.

**Table 2:** Differences in depression symptom scores of nurses among different departments.

Variable	Category	*Z*	*p*
Department	Outpatient and Other Departments vs. Internal Medicine, Gynaecology and Paediatrics	3.364	<0.001
	Outpatient and Other Departments vs. Emergency Department	−3.319	<0.001
	Outpatient and Other Departments vs. Intensive Care Unit	−4.583	<0.001
	Internal Medicine, Gynaecology and Paediatrics vs. Emergency Department	−1.420	0.156
	Internal Medicine, Gynaecology and Paediatrics vs. Intensive Care Unit	−2.906	0.004
	Emergency Department vs. Intensive Care Unit	−1.007	0.314

### 3.2 Scores for each main variable

The scores for each scale are presented in Table 3 The mean night shift workload intensity score was 54.51 ± 27.49, and the mean depression symptom score was 9.38 ± 5.42, with a detection rate of 50.81%. The PSQI total score averaged 7.22 ± 3.34, with a sleep disturbance detection rate (PSQI > 7) of 30.94%. Scores for subjective sleep quality, sleep onset latency, and daytime functional impairment were notably elevated. The SADS total score was 14.65 ± 6.79, with social avoidance and social distress scores of 7.75 ± 3.46 and 6.90 ± 3.75, respectively. The total FOMO score was 21.88 ± 8.08, predominantly driven by the fear of missing situations (11.95 ± 4.02).

**Table 3:** Scores for main variables.

Scale	Dimension (score range)	*M* ± *SD*
Night shift intensity	Score	54.51 ± 27.49
CESD-10	Score (0–30)	9.38 ± 5.42
PSQI	Score (0–21)	7.22 ± 3.34
	Subjective sleep quality (0–3)	1.62 ± 0.63
	Duration of sleep (0–3)	1.07 ± 0.82
	Sleep efficiency (0–3)	0.56 ± 0.81
	Sleep disorder (0–3)	0.44 ± 0.54
	Bedtime (0–3)	1.59 ± 1.14
	Hypnotic drug use (0–3)	0.27 ± 0.69
	Daytime dysfunction (0–3)	1.67 ± 0.94
SADS	Score (0–28)	14.65 ± 6.79
	Social avoidance (0–14)	7.75 ± 3.46
	Social anxiety (0–14)	6.90 ± 3.75
FOMOS	Score (8–40)	21.88 ± 8.08
	Fear of Missing Out Information (4–20)	9.93 ± 4.44
	Fear of Missing Out on Situations (4–20)	11.95 ± 4.02

CESD-10, Centre for Epidemiologic Studies Depression Scale-10; PSQI, Pittsburgh Sleep Quality Index; SADS, Social Avoidance and Distress Scale; FOMOS, Fear of Missing Out Scale.

### 3.3 Correlations between depressive symptoms and independent variables

As shown in Table 4, Spearman correlation analysis revealed significant positive correlations between night shift intensity and depression (*r* = 0.335, *p* < 0.01), SAD (*r* = 0.184, *p* < 0.01), FOMO (*r* = 0.301, *p* < 0.01), and sleep disturbance (*r* = 0.263, *p* < 0.01).

**Table 4:** Correlation analysis between night shift work intensity and depressive symptoms, sleep disturbances, social avoidance and distress, and fear of missing out among nurses (*N* = 614).

Variable	1	2	3	4	5
1. Night shift workload	1				
2. Sleep disturbances	0.384**	1			
3. Social avoidance and distress	0.184**	0.361**	1		
4. Fear of missing out	0.301**	0.249**	0.268**	1	
5. Depressive symptoms	0.335**	0.384**	0.451**	0.389**	1

Values are Spearman’s rank correlation coefficients. **indicates *p* < 0.01.

### 3.4 Mediation effect of sleep disorders, SAD, and FOMO on depressive symptoms through night shift work intensity

**Figure 1:**
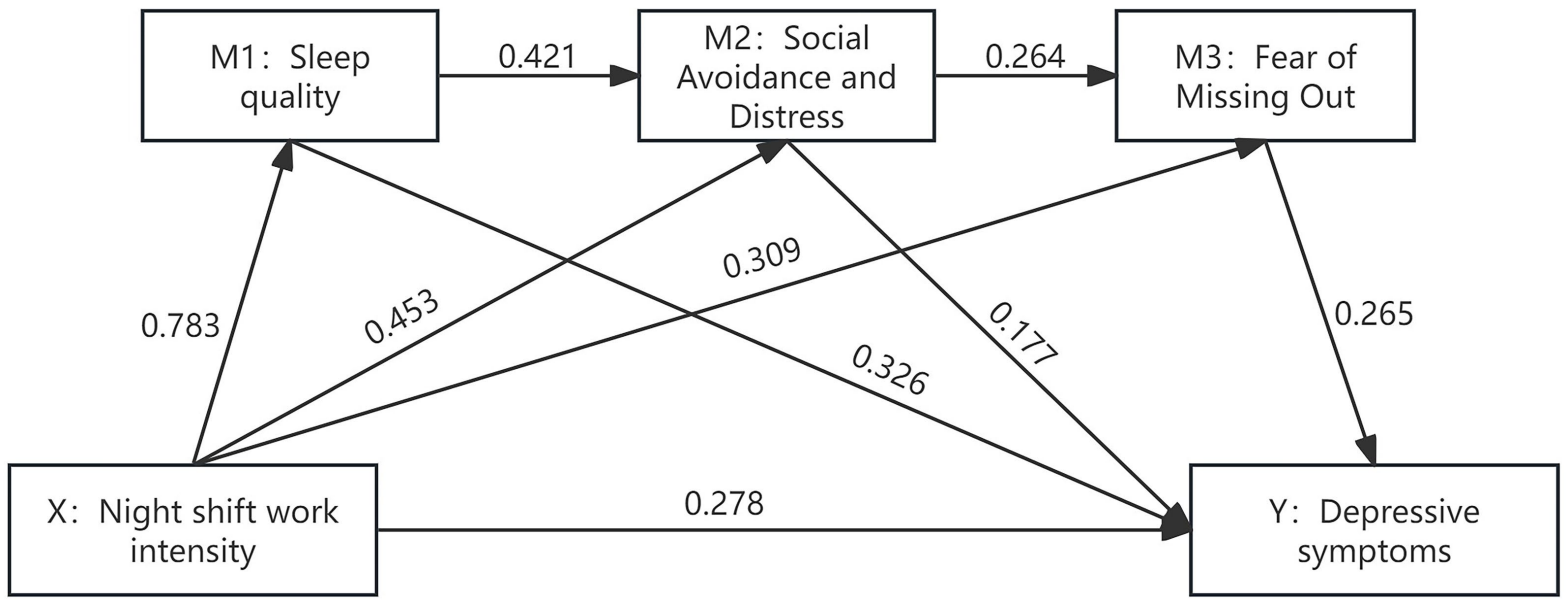
Mediation model diagram of sleep disturbances, social avoidance and distress, and fear of missing out in the relationship between night shift work intensity and depressive symptoms among nurses (*N* = 614).

Mediation analysis was conducted using the PROCESS macro (Model 6) in SPSS 25.0. Figure 1, Table 5 show that the total effect of night shift work intensity on depressive symptoms was significant (*β* = 0.1314, *p* < 0.001). This result indicates that higher night shift work intensity correlates with more severe depressive symptoms. The direct effect remained significant after incorporating all mediating variables (*β* = 0.0416, 95% *CI*: 0.0351–0.0481, *p* < 0.001), suggesting a partial direct pathway between night shift workload intensity and depressive symptoms. Overall, the mediation effect was significant, with a total indirect effect of 0.0898 (*p* < 0.001), accounting for 68.34% of the total effect. Specific indirect path analysis revealed: the mediating effect through sleep disturbance (M1) was 0.0381 (95% *CI*: 0.0307–0.0459), accounting for 29.00% of the total indirect effect; the effect via SAD (M2) was 0.0120 (95% *CI*: 0.0078–0.0168), accounting for 9.13%; and via FOMO (M3) was 0.0122 (95% *CI*: 0.0080–0.0170), accounting for 9.28%. The complete chain pathway (Night-shift work intensity → Sleep disturbance → Social avoidance → FOMO → Depressive symptoms) had an effect value of 0.0034 (95% *CI*: 0.0019–0.0055), accounting for 2.59%. Further structural equation modelling tested the stability of the model, revealing that the mediation model met fit criteria, as shown in Table 6.

**Table 5:** Results of the mediating effect analysis of sleep disturbances, social avoidance and distress, and fear of missing out in the relationship between night shift work intensity and depressive symptoms among nurses.

Paths	Effects	Boot SE	LLCI	ULCI	Effect proportion	*p*
Total effect	0.1314	0.0029	0.1257	0.1370	100%	<0.001
Direct effect	0.0416	0.0033	0.0351	0.0481	31.66%	<0.001
Total indirect effect	0.0898	0.0036	0.0827	0.0966	68.34%	<0.001
Indirect effect: night shift workload → sleep disturbances → depressive symptoms	0.0381	0.0038	0.0307	0.0459	29.00%	–
Indirect effect: night shift workload → SAD → depressive symptoms	0.0120	0.0023	0.0078	0.0168	9.13%	–
Indirect effect: night shift workload → FOMO → depressive symptoms	0.0122	0.0023	0.0080	0.0170	9.28%	–
Indirect effect: night shift workload → sleep disturbances → SAD → depressive symptoms	0.0087	0.0017	0.0055	0.0124	6.62%	–
Indirect effect: night shift workload → sleep disturbances → SAD → FOMO → depressive symptoms	0.0034	0.0009	0.0019	0.0055	2.59%	–

The table showed the total effect, direct effect, indirect effect, and Bootstrap path effect test. SAD, social avoidance and distress; FOMO, fear of missing out; SE, standard error; LLCI, lower limit of the confidence interval; ULCI, upper limit of the confidence interval. – indicates missing values.

**Table 6:** Goodness of fit of structural equation modeling.

Fit indices	Model fit	Reference value
*χ*^2^/df	2.71	<3
CFI	0.958	>0.90
TLI	0.941	>0.90
RMSEA	0.053	<0.08
SRMR	0.038	<0.08

*χ*^2^/df, chi-square to degrees of freedom ratio; CFI, comparative fit index; TLI, Tucker-Lewis index; RMSEA, root mean square error of approximation; SRMR, standardized root mean square residual.

## 4 Discussion

This study represents the first investigation into the association between sleep disturbances, SAD and FOMO in relation to night shift work and depressive symptoms among nurses. Findings indicate: (1) Currently, clinical nurses in our country face heavy workloads during night shifts and have a relatively high incidence of depression. (2) Night shift work intensity exhibits significant positive correlations with nurses’ depressive symptoms, sleep disturbances, social avoidance and distress, and fear of missing out; (3) Sleep disturbances, SAD, and FOMO partially mediated the relationship between night shift workload intensity and depressive symptoms among nurses; (4) Nurses with lower educational attainment, higher monthly frequency of night shifts, poorer sleep quality, working in emergency departments or intensive care units, and higher levels of SAD and FOMO were more prone to depressive symptoms, consistent with previous research findings (7, 39, 40).

Mental health issues among nurses are increasingly prominent, representing a critical public health concern that affects both healthcare service quality and occupational wellbeing. Globally, the prevalence of depression among healthcare workers is approximately 37% (41). In this study, nurses exhibited a mean depression score of 9.38 ± 5.42, with a detection rate of 50.81%, exceeding the global prevalence among healthcare workers and aligning with previous studies. For instance, Li and Fu (8) reported a mean depression score of 9.20 ± 5.40 among nurses, with a prevalence of 45.9%, while a survey in Hainan Province, China, indicated a depression prevalence of 42.7% among healthcare workers (42). These findings suggest that Chinese nurses face particularly severe mental health challenges. One contributing factor is the relative shortage of nursing personnel: according to World Health Statistics 2022, China has approximately 3.7 registered nurses per 1,000 inhabitants, which, although improved, remains substantially lower than in developed countries such as the United States (≈15.7), Japan (≈12.7), and Germany (≈13.5) (43). This shortage results in prolonged excessive workloads, including heavy clinical duties, administrative responsibilities, frequent emergencies, continuous shifts, and intensive night duties, which disrupt circadian rhythms and increase the prevalence of sleep disorders, compromise immunity, and lead to chronic fatigue (44). As healthcare professionals with the closest and longest-duration patient contact, nurses undertake multiple responsibilities, including basic care, condition monitoring, health education, psychological support, emergency resuscitation, and high-risk procedures, within a high-stress environment demanding significant emotional investment (45, 46). Compounding these pressures, occupational protection mechanisms in China remain inadequate, with frequent exposure to hazards such as needle stick injuries, infectious agents, and musculoskeletal strain, exacerbating both physical and mental attrition (47). Public recognition of nursing remains limited, with nurses often perceived as “assistants to doctors” rather than independent specialists, resulting in low occupational dignity and societal recognition (48). Consequently, nurses are frequently targeted as outlets for patients’ and families’ negative emotions, with incidents of verbal and even physical altercations being common; surveys indicate that approximately 86.7% of Chinese nurses have experienced workplace violence, severely undermining psychological safety and professional belonging (43). The cumulative effect of these multiple pressures has led to alarming mental health outcomes, with nurses exhibiting higher rates of depression, anxiety, and occupational burnout compared to the general population (49). In summary, the high prevalence of mental health issues among Chinese nurses is driven by interrelated factors including human resource shortages, excessive workloads, insufficient social support, and inadequate professional recognition. Urgent measures are needed to establish a systematic occupational health protection framework, including scientific staffing allocation, optimized shift scheduling, strengthened psychological support, enhanced occupational safeguards, and improved societal recognition, to promote the sustainable development of the nursing workforce and continuous improvement in healthcare service quality.

This study revealed that the intensity of night shift work among nurses significantly and positively predicted depressive symptoms. Greater night shift workload was correlated with higher depression risk, a finding consistent with previous research (45–47). A prospective cohort study using UK Biobank data by Chen et al. (50) demonstrated that shift workers undertaking more than eight night shifts per month experienced a 40% increased risk of depression. That study further indicated that night shift work increased depression risk regardless of genetic predisposition. Li and Fu (8) similarly observed that nurses working a greater number of night shifts per week and longer hours exhibited greater susceptibility to depressive symptoms. A survey of 9,789 female nurses in South Korea showed that night shift nurses faced a 1.52-fold higher risk of depressive symptoms compared to non-night shift nurses (51). A meta-analysis similarly reported that night shift workers had a 1.43-fold higher risk of depression compared to non-night shift workers (52). These studies collectively support the present findings, confirming a significant positive correlation between night shift work and depressive symptoms among nurses. Although the underlying mechanisms linking night shift work to increased depression risk remain incompletely elucidated, existing research suggests several potential pathways. Hospitals operate around the clock, requiring nurses to undertake prolonged night shifts and frequently adapt to irregular schedules. This misalignment between intrinsic physiological rhythms and external demands severely disrupts sleep–wake cycles, work-life balance, and social functioning, thereby facilitating the onset of depressive symptoms (12). Night shift work disrupts the normal light-melatonin rhythm, as nighttime illumination suppresses pineal melatonin secretion while daytime sleep patterns further disturb circadian rhythmicity (53). Studies show that night work reduces daytime sunlight exposure, with approximately 58% of shift nurses exhibiting melatonin concentrations below the health threshold (54). Melatonin not only regulates sleep but also modulates mood; its depletion weakens the brain’s capacity to regulate negative emotions, increasing susceptibility to depression (53). Additionally, night shift work activates the hypothalamic–pituitary–adrenal axis, triggering chronic stress responses (55). Elevated morning cortisol levels have been observed in some shift workers, particularly younger nurses, prolonging physiological arousal and impairing hippocampal function. Depressive states can further exacerbate morning cortisol elevation, creating a mutually reinforcing vicious cycle (56). Night shift work may also reduce *α*-amylase levels, a biomarker of sympathetic nervous system activity regulated by the autonomic nervous system. Lower α-amylase levels indicate autonomic imbalance and inadequate physiological arousal, impairing emotional regulation and stress adaptation, thereby exacerbating emotional fatigue and depressive tendencies (57).

The findings of this study indicate that sleep disturbances partially mediate the relationship between night shift workload intensity and depressive symptoms among nurses, consistent with findings reported by Dai et al. (30). Their results showed a significantly higher prevalence of depression among night shift nurses compared to day shift nurses, primarily due to the decline in sleep quality caused by night work. Among the mediators examined, sleep disturbance exhibited the strongest mediating effect. Thus, improving sleep quality may be a key intervention target for alleviating depressive symptoms in nurses, consistent with previous research (58, 59). Sun et al. (59) demonstrated a bidirectional positive correlation between sleep quality and depression: poorer sleep quality correlates with more severe depression, and vice versa. Okechukwu et al. (60) and Brown et al. (61) further emphasized that sleep quality plays a critical role in the psychological health impacts of night shift work. Moreover, the effect of poor sleep on depression is greater than the reverse effect, with severe sleep disturbances often underlying the adverse psychological health effects of night shifts. Additionally, a prospective cohort study demonstrated that employees with irregular sleep patterns and sustained night shift work face an increased risk of depression (62). These studies collectively reinforce the present findings that sleep disturbances mediate the relationship between night shift work and depressive symptoms among nurses. Sleep deprivation following night shifts is a prevalent challenge among nurses (63). Sleep plays a pivotal role in restoring physiological functions, regulating emotional experiences, and coping with psychological stress. Adequate sleep quality reduces the adverse effects of stress on mental health and supports adaptive processing of emotional stimuli within the brain (64). Night shifts disrupt normal circadian rhythms, suppress melatonin secretion, and interfere with sleep initiation and maintenance (65). Circadian rhythm disruption also causes autonomic nervous system imbalance, characterized by persistently heightened sympathetic activity and suppressed parasympathetic function. This state of “persistent alertness” makes relaxation difficult, further disrupting sleep architecture and impairing emotional regulation (66). Such autonomic imbalance exacerbates anxiety and irritability and disrupts functional integration within emotional centers, increasing sensitivity to negative emotions (67). Chronic sleep deprivation additionally diminishes the prefrontal cortex’s capacity to regulate amygdala-mediated emotions, heightening vulnerability to stressors (68), and elevates pro-inflammatory factors such as IL-6 and TNF-α, inducing chronic low-grade inflammation that disrupts neurotransmitter metabolism and neural plasticity, thereby further promoting depressive onset (69, 70). Consequently, sleep management for night shift workers warrants attention. Optimizing shift patterns and implementing targeted sleep interventions can provide early disruption of depressive risk pathways, while integrating sleep health into routine occupational assessments allows timely identification and intervention for sleep disturbances, reducing the risk of depression onset.

This study found that sleep disturbances and SAD mediated the relationship between night shift work intensity and depressive symptoms among nurses. Prolonged night shifts disrupt normal circadian rhythms, suppress melatonin secretion, and trigger or exacerbate sleep disturbances. Sustained poor sleep quality not only directly impairs emotional stability but also diminishes the prefrontal cortex’s regulatory control over the limbic system, increasing sensitivity to perceived social threats and fostering tension, anxiety about negative evaluation, and heightened self-consciousness during interpersonal interactions, thereby promoting the development of SAD (71). Nurses experiencing persistent fatigue, distractibility, and emotional instability due to sleep deprivation often reduce communication with colleagues, friends, and even family members, leading to decreased participation in group activities and social interactions and resulting in social isolation (72). Although such avoidance may temporarily alleviate anxiety, the prolonged absence of positive social feedback and support intensifies feelings of loneliness, self-denial, and worthlessness, becoming a significant psychological precursor to depression (73). Thus, night shifts not only directly disrupt circadian rhythms to affect mental health but also gradually accumulate psychological risks through sleep disturbances and social avoidance, ultimately driving the onset and progression of depressive symptoms. Future psychological interventions for night-shift nurses should extend beyond isolated symptom management, prioritizing comprehensive support that spans sleep improvement and social function restoration. Recommended strategies include optimizing organizational shift patterns to minimize frequent rotations, ensuring adequate rest periods, and fostering supportive work environments. Concurrently, peer support groups and mobile-platform-based mindfulness training or cognitive behavioral self-help tools can help nurses enhance emotional regulation, rebuild positive social connections, and effectively interrupt the risk pathway from sleep disturbance through SAD to depression.

The findings of this study indicate that sleep disturbances, SAD, and FOMO partially mediated the relationship between night shift work intensity and depressive symptoms among nurses. Prolonged night shift rotations disrupt nurses’ ability to maintain normal social rhythms, as most individuals engage in daytime social activities while night shift nurses are often resting or preparing for work. This asynchronous lifestyle frequently causes them to miss family gatherings, social interactions with friends, and colleague exchanges, gradually fostering a sense of social disconnection (23). Within this context, individuals become more prone to social avoidance behaviors, manifested as apprehension toward interpersonal interactions, withdrawal, and emotional avoidance, which may escalate into persistent social anxiety and trigger FOMO (74). FOMO refers to an anxious preoccupation with others’ positive social experiences, characterized by intense cognitive and emotional reactions centered on the fear of exclusion (19). In today’s hyper-connected multimedia era, the ubiquity of social media enables real-time access to others’ lives, amplifying night shift nurses’ perception of social loss (75). After enduring sleep disturbances and social isolation, nurses often perceive others’ vibrant experiences, such as travel, social gatherings, and career achievements, through social media, reinforcing the cognitive bias that they are missing out on important experiences (76). Research indicates that FOMO is closely associated with loneliness and emotional dysregulation and functions as a psychological amplifier, transforming physiological stressors (night shifts, sleep disorders) and social alienation (social avoidance) into persistent internalized anxiety, which can further evolve into depressive symptoms (31). Together, these results demonstrate that depression does not result from a single factor but arises through the dynamic interplay of multi-stage psychological processes. Therefore, it is recommended to develop a phased psychological intervention system targeting sleep and social avoidance immediately after nurses complete their night shift, thereby interrupting the step-by-step transmission of negative affect. Concurrently, a digital health-support platform should be established, integrating sleep management, cognitive regulation, and social-connection functions to provide comprehensive, continuous mental health support for nurses working night shifts.

## 5 Strengths and limitations

This study is the first to incorporate sleep disturbances, SAD, and FOMO into a single model, validating their mediating role between night shift work and depression among nurses. It identifies a multi-layered pathway linking occupational exposure to psychological issues, specifically focusing on China’s clinical nursing population to address practical demands for mental health interventions among frontline staff. Nevertheless, this study has several limitations. Firstly, the cross-sectional design prevents causal inference. Secondly, reliance on self-reported data may introduce recall or social desirability biases; objective measures, such as sleep monitoring, physiological indicators, or multi-source assessments, were not included. Thirdly, although the model identified three mediating pathways, night shift intensity continued to have a significant direct effect on depressive symptoms (approximately 52.6% of the total effect). Thus, other unmeasured factors, such as job satisfaction, chronic illnesses, personality traits (e.g., neuroticism), occupational burnout, and departmental staffing levels, may also influence depression risk. These factors might relate to night shift arrangements or directly affect mental health, yet were not examined in this analysis. Finally, the study sample included nurses from only one tertiary hospital in Haikou City, Hainan Province, with 94% being female, limiting geographical and sample representativeness. Caution should therefore be exercised when extrapolating the results. Future research should utilize objective metrics within a multicentre, large-sample longitudinal design, include nurses from diverse regions and of different genders, and integrate comprehensive psychological, organisational, and health-related variables. Such approaches would facilitate dynamic tracking of night shift mechanisms affecting mental health, thereby enhancing the generalisability of the findings.

## 6 Conclusion

This study found that night shift workload intensity positively predicted depressive symptoms among nurses. Additionally, sleep disturbances, SAD, and FOMO partially mediated the relationship between night shift workload intensity and depressive symptoms among nurses. Future interventions should comprehensively address sleep management, social support, and digital psychological adaptation for night shift nurses to mitigate depressive symptoms.

## Data Availability

The original contributions presented in the study are included in the article/Supplementary material, further inquiries can be directed to the corresponding authors.

## Footnotes

1
https://www.wenjuan.com/

## References

[1] Cai Y Li Q Cao T Wan Q. Nurses' work engagement: the influences of ambidextrous leadership, clinical nurse leadership and workload. J Adv Nurs. (2023) 79:1152–61. doi: 10.1111/jan.15086, 34723406

[2] Fu C Lv X Cui X Huang M Cao F. The association between fear of future workplace violence and depressive symptoms among nurses based on different experiences of workplace violence: a cross-sectional study. BMC Nurs. (2023) 22:123. doi: 10.1186/s12912-023-01265-1, 37061670 PMC10105151

[3] Alhowaymel FM Saleh MSM Taref NN Abd-Elhamid ZN Abaoud AF Alenezi A . Empowering future nurses: enhancing self-efficacy, satisfaction, and academic achievement through talent management educational intervention. BMC Nurs. (2025) 24:875. doi: 10.1186/s12912-025-03512-z, 40624493 PMC12235881

[4] Cui S Zhang L Yan H Shi Q Jiang Y Wang Q . Experiences and psychological adjustments of nurses who voluntarily supported covid-19 patients in Hubei province, China. Psychol Res Behav Manag. (2020) 13:1135–45. doi: 10.2147/PRBM.S283876, 33312005 PMC7727274

[5] Cabrera-Aguilar E Zevallos-Francia M Morales-García M Ramírez-Coronel AA Morales-García SB Sairitupa-Sanchez LZ . Resilience and stress as predictors of work engagement: the mediating role of self-efficacy in nurses. Front Psych. (2023) 14:1202048. doi: 10.3389/fpsyt.2023.1202048, 37649562 PMC10464840

[6] Zhou Y Gao W Li H Yao X Wang J Zhao X. Network analysis of resilience, anxiety and depression in clinical nurses. BMC Psychiatry. (2024) 24:719. doi: 10.1186/s12888-024-06138-8, 39438840 PMC11520162

[7] Xie N Qin Y Wang T Zeng Y Deng X Guan L. Prevalence of depressive symptoms among nurses in China: a systematic review and meta-analysis. PLoS One. (2020) 15:e235448. doi: 10.1371/journal.pone.0235448, 32634150 PMC7340293

[8] Li C Fu C. Workplace violence and depressive symptoms: the mediating role of fear of future workplace violence and burnout among Chinese nurses. BMC Psychiatry. (2024) 24:379. doi: 10.1186/s12888-024-05827-8, 38773476 PMC11110276

[9] Dall'Ora C Ejebu O Ball J Griffiths P. Shift work characteristics and burnout among nurses: cross-sectional survey. Occup Med (Lond). (2023) 73:199–204. doi: 10.1093/occmed/kqad046, 37130349 PMC10195190

[10] Zhang H Wang J Zhang S Tong S Hu J Che Y . Relationship between night shift and sleep problems, risk of metabolic abnormalities of nurses: a 2 years follow-up retrospective analysis in the National Nurse Health Study (NNHS). Int Arch Occup Environ Health. (2023) 96:1361–71. doi: 10.1007/s00420-023-02014-2, 37874403 PMC10635907

[11] Li Y Wang Y Lv X Li R Guan X Li L . Effects of factors related to shift work on depression and anxiety in nurses. Front Public Health. (2022) 10:926988. doi: 10.3389/fpubh.2022.926988, 35910870 PMC9326492

[12] Okechukwu CE Colaprico C Di Mario S Oko-Oboh AG Shaholli D Manai MV . The relationship between working night shifts and depression among nurses: a systematic review and meta-analysis. Healthcare. (2023) 11:937. doi: 10.3390/healthcare11070937, 37046864 PMC10094007

[13] Vieira LMSM Mininel VA Sato TDO. Sleep quality as a mediator of burnout, stress and multisite musculoskeletal pain in healthcare workers: a longitudinal study. Healthcare. (2023) 11:2476. doi: 10.3390/healthcare11182476, 37761673 PMC10531134

[14] Pan Y Wang X Jin W. Risk of compassion fatigue among emergency department nurses: a systematic review and meta-analysis. BMC Emerg Med. (2025) 25:155. doi: 10.1186/s12873-025-01314-9, 40817038 PMC12357388

[15] Li X Shen H Kong H Xie J. Autistic traits predict social avoidance and distress: the chain mediating role of perceived stress and interpersonal alienation. Scand J Psychol. (2023) 64:802–9. doi: 10.1111/sjop.12946, 37345676

[16] Li S Kong K Zhang K Niu H. The relation between college students’ neuroticism and loneliness: the chain mediating roles of self-efficacy, social avoidance and distress. Front Psychol. (2023) 14:1124588. doi: 10.3389/fpsyg.2023.1124588, 37138990 PMC10149762

[17] Yuan Y Jiang S Yan S Chen L Zhang M Zhang J . The relationship between depression and social avoidance of college students: a moderated mediation model. J Affect Disord. (2022) 300:249–54. doi: 10.1016/j.jad.2021.12.119, 34979184

[18] Wang Y Liu M Yang F Chen H Wang Y Liu J. The associations of socioeconomic status, social activities, and loneliness with depressive symptoms in adults aged 50 years and older across 24 countries: findings from five prospective cohort studies. Lancet Healthy Longev. (2024) 5:100618. doi: 10.1016/j.lanhl.2024.07.001, 39208829

[19] Mert M Tengilimoğlu D. The mediating role of FOMO and the moderating role of narcissism in the impact of social exclusion on compulsive buying: a cross-cultural study. Psicol Reflex Crit. (2023) 36:33. doi: 10.1186/s41155-023-00274-y, 37934364 PMC10630266

[20] Zhang Y Shang S Tian L Zhu L Zhang W. The association between fear of missing out and mobile phone addiction: a meta-analysis. BMC Psychol. (2023) 11:338. doi: 10.1186/s40359-023-01376-z, 37848985 PMC10580531

[21] Gao B Shen Q Luo G Xu Y. Why mobile social media-related fear of missing out promotes depressive symptoms? The roles of phubbing and social exclusion. BMC Psychol. (2023) 11:189. doi: 10.1186/s40359-023-01231-1, 37386513 PMC10311784

[22] Mercan Işik C öztürk M. The relationship between chronotype characteristics and fear of missing out, phubbing, sleep quality and social jetlag in medical students. Chronobiol Int. (2024) 41:1340–50. doi: 10.1080/07420528.2024.2416986, 39431646

[23] Nilsson T Lashari A Gustavsson P Härmä M Bigert C Bodin T . Night and shift work and incidence of physician-diagnosed sleep disorders in nursing staff: a prospective cohort study. Int J Nurs Stud. (2025) 164:105017. doi: 10.1016/j.ijnurstu.2025.105017, 39929033

[24] Xu Y Chen Q Tian Y. The impact of problematic social media use on inhibitory control and the role of fear of missing out: evidence from event-related potentials. Psychol Res Behav Manag. (2024) 17:117–28. doi: 10.2147/PRBM.S441858, 38223309 PMC10787569

[25] Elhai JD Gallinari EF Rozgonjuk D Yang H. Depression, anxiety and fear of missing out as correlates of social, non-social and problematic smartphone use. Addict Behav. (2020) 105:106335. doi: 10.1016/j.addbeh.2020.106335, 32062337

[26] Monroe SM Simons AD. Diathesis-stress theories in the context of life stress research: implications for the depressive disorders. Psychol Bull. (1991) 110:406–25. doi: 10.1037/0033-2909.110.3.406, 1758917

[27] Katerndahl DA Parchman M. The ability of the stress process model to explain mental health outcomes. Compr Psychiatry. (2002) 43:351–60. doi: 10.1053/comp.2002.34626, 12216010

[28] DI Milia L Bjorvatn B. The relationship between shift work, sleep, and work hours on wellbeing. Ind Health. (2025) 63:148–55. doi: 10.2486/indhealth.2024-0088, 39155078 PMC11995149

[29] Hyndych A El-Abassi R Mader ECJ. The role of sleep and the effects of sleep loss on cognitive, affective, and behavioral processes. Cureus. (2025) 17:e84232. doi: 10.7759/cureus.84232, 40525051 PMC12168795

[30] Dai C Qiu H Huang Q Hu P Hong X Tu J . The effect of night shift on sleep quality and depressive symptoms among Chinese nurses. Neuropsychiatr Dis Treat. (2019) 15:435–40. doi: 10.2147/NDT.S190689, 30799922 PMC6369837

[31] Qutishat M Al Sabei S. Relationship between burnout and fear of missing out among nurses in Oman: implication for nursing practice. J Educ Health Promot. (2024) 13:495. doi: 10.4103/jehp.jehp_429_24, 39850301 PMC11756687

[32] Alreshidi SM Rayani AM. The correlation between night shift work schedules, sleep quality, and depression symptoms. Neuropsychiatr Dis Treat. (2023) 19:1565–71. doi: 10.2147/NDT.S421092, 37440839 PMC10335288

[33] Andresen EM Malmgren JA Carter WB Patrick DL. Screening for depression in well older adults: evaluation of a short form of the CES-D (Center for Epidemiologic Studies Depression Scale). Am J Prev Med. (1994) 10:77–84. doi: 10.1016/S0749-3797(18)30622-6, 8037935

[34] Buysse DJ Reynolds CFR Monk TH Berman SR Kupfer DJ. The Pittsburgh Sleep Quality Index: a new instrument for psychiatric practice and research. Psychiatry Res. (1989) 28:193–213. doi: 10.1016/0165-1781(89)90047-4, 2748771

[35] Chen Z He K Chen Y Zhang X Ye Z Xie C . Sleep quality mediates the effect of medical social support on depression symptoms in patients with HIV/Aids. BMC Public Health. (2024) 24:1429. doi: 10.1186/s12889-024-18174-w, 38807089 PMC11134677

[36] Lu Q Wang M Zuo Y Tang Y Zhang R Zhang J. Construction and verification of a risk prediction model of psychological distress in psychiatric nurses. BMC Nurs. (2025) 24:161. doi: 10.1186/s12912-025-02796-5, 39934812 PMC11817827

[37] Wang X Wang X Ma H. Manual of Mental Health Rating Scales. Beijing: China Mental Health Magazine. (1999) 241–244.,

[38] Wu W Zhang J Jo N. Fear of missing out and online social anxiety in university students: mediation by irrational procrastination and media multitasking. Behav Sci (Basel). (2025) 15:84. doi: 10.3390/bs15010084, 39851888 PMC11762956

[39] Zhou Y Wang S Liu M Gan G Qin N Luo X . The role of sleep quality and perceived stress on depressive symptoms among tertiary hospital nurses: a cross-sectional study. BMC Psychiatry. (2023) 23:416. doi: 10.1186/s12888-023-04936-0, 37308915 PMC10258928

[40] Gong Y Han T Yin X Yang G Zhuang R Chen Y . Prevalence of depressive symptoms and work-related risk factors among nurses in public hospitals in southern China: a cross-sectional study. Sci Rep. (2014) 4:7109. doi: 10.1038/srep07109, 25427988 PMC5384112

[41] Saragih ID Tonapa SI Saragih IS Advani S Batubara SO Suarilah I . Global prevalence of mental health problems among healthcare workers during the COVID-19 pandemic: a systematic review and meta-analysis. Int J Nurs Stud. (2021) 121:104002. doi: 10.1016/j.ijnurstu.2021.104002, 34271460 PMC9701545

[42] Lu G Xiao S He J Xie W Ge W Meng F . Prevalence of depression and its correlation with anxiety, headache and sleep disorders among medical staff in the Hainan province of China. Front Public Health. (2023) 11:1122626. doi: 10.3389/fpubh.2023.1122626, 37441641 PMC10333496

[43] Wang B Yang W Wang Y Chen X Liu D Yin B . Current situation and related factors of fatigue among doctors and nurses in tertiary general hospitals in Northeast China. Sci Rep. (2025) 15:9548. doi: 10.1038/s41598-025-87400-1, 40108223 PMC11923294

[44] Sancini A Ciarrocca M Capozzella A Corbosiero P Fiaschetti M Caciari T . Shift and night work and mental health. G Ital Med Lav Ergon. (2012) 34:76–84. 22697038

[45] Li Y Wu J Liu X Zhang J Zhong X He L. Latent profile analysis and influence factors study of presenteeism among ICU nurses in China. Front Psychol. (2023) 14:1259333. doi: 10.3389/fpsyg.2023.1259333, 38023026 PMC10644221

[46] Alimoradi Z Jafari E Lin C Rajabi R Marznaki ZH Soodmand M . Estimation of moral distress among nurses: a systematic review and meta-analysis. Nurs Ethics. (2023) 30:334–57. doi: 10.1177/09697330221135212, 36704986 PMC9902807

[47] Muthelo L Sinyegwe NF Phukubye TA Mbombi MO Ntho TA Mothiba TM. Prevalence of work-related musculoskeletal disorders and its effects amongst nurses in the selected intellectual disability unit of the Limpopo province. Healthcare (Basel). (2023) 11:777. doi: 10.3390/healthcare11050777, 36900781 PMC10000717

[48] Jiang H Huang N Tian W Shi S Yang G Pu H. Factors associated with post-traumatic stress disorder among nurses during COVID-19. Front Psychol. (2022) 13:745158. doi: 10.3389/fpsyg.2022.745158, 35173657 PMC8841878

[49] Navarro-Abal Y Climent-Rodríguez JA Vaca-Acosta RM Fagundo-Rivera J Gómez-Salgado J García-Iglesias JJ. Workplace violence: impact on the commitment and involvement of nurses at work. J Nurs Manag. (2023) 2023:9987092. doi: 10.1155/2023/9987092, 40225620 PMC11918937

[50] Chen Y Yang H Zhang Y Zhou L Lin J Wang Y. Night shift work, genetic risk, and the risk of depression: a prospective cohort study. J Affect Disord. (2024) 354:735–42. doi: 10.1016/j.jad.2024.03.134, 38548197

[51] Lee HY Kim MS Kim O Lee I Kim H. Association between shift work and severity of depressive symptoms among female nurses: the Korea nurses' health study. J Nurs Manag. (2016) 24:192–200. doi: 10.1111/jonm.12298, 25950801

[52] Lee A Myung SK Cho JJ Jung YJ Yoon JL Kim MY. Night shift work and risk of depression: meta-analysis of observational studies. J Korean Med Sci. (2017) 32:1091–6. doi: 10.3346/jkms.2017.32.7.1091, 28581264 PMC5461311

[53] Boivin DB Boudreau P Kosmadopoulos A. Disturbance of the circadian system in shift work and its health impact. J Biol Rhythm. (2022) 37:3–28. doi: 10.1177/07487304211064218, 34969316 PMC8832572

[54] Vivarelli S Italia S Teodoro M Pollicino M Vitello C De Vita A . Salivary biomarkers analysis and neurobehavioral assessment in nurses working rotation shifts: a pilot study. Int J Environ Res Public Health. (2023) 20:5376. doi: 10.3390/ijerph20075376, 37047991 PMC10094107

[55] Zhang S Wang Y Zhu Y Li X Song Y Yuan J. Rotating night shift work, exposure to light at night, and glomerular filtration rate: baseline results from a Chinese occupational cohort. Int J Environ Res Public Health. (2020) 17:9035. doi: 10.3390/ijerph17239035, 33291553 PMC7730862

[56] Yang Z Black K Ohman-Strickland P Graber JM Kipen HM Fang M . Disruption of central and peripheral circadian clocks and circadian controlled estrogen receptor rhythms in night shift nurses in working environments. FASEB J. (2024) 38:e23719. doi: 10.1096/fj.202302261RR, 38837828 PMC11884403

[57] Barth J Greene JA Goldstein J Sibley A. Adverse health effects related to shift work patterns and work schedule tolerance in emergency medical services personnel: a scoping review. Cureus. (2022) 14:e23730. doi: 10.7759/cureus.23730, 35509733 PMC9060748

[58] Pandi-Perumal SR Monti JM Burman D Karthikeyan R Bahammam AS Spence DW . Clarifying the role of sleep in depression: a narrative review. Psychiatry Res. (2020) 291:113239. doi: 10.1016/j.psychres.2020.113239, 32593854

[59] Sun X Liu B Liu S Wu DJH Wang J Qian Y . Sleep disturbance and psychiatric disorders: a bidirectional Mendelian randomisation study. Epidemiol Psychiatr Sci. (2022) 31:e26. doi: 10.1017/S2045796021000810, 35465862 PMC9069588

[60] Okechukwu CE Griffiths MD Carta MG Nwobodo E Shariful Islam SM Forbes M . Biological and practical considerations regarding circadian rhythm and mental health relationships among nurses working night shifts: a narrative review and recommendations. Riv Psichiatr. (2022) 57:67–79. doi: 10.1708/3790.37738, 35426425

[61] Brown JP Martin D Nagaria Z Verceles AC Jobe SL Wickwire EM. Mental health consequences of shift work: an updated review. Curr Psychiatry Rep. (2020) 22:7. doi: 10.1007/s11920-020-1131-z, 31955278

[62] Liu B Jia C. Shift work schedule and sleep patterns in relation to incident depression: evidence from a prospective cohort study. Psychiatry Res. (2023) 321:115076. doi: 10.1016/j.psychres.2023.115076, 36739727

[63] Yi X Jing C Meimei M Jianhui X Jihong H Ding X . Acute stress reaction, depression anxiety stress, and job withdrawal behavior in non-frontline pediatric nurses during the pandemic: a cross-sectional study. Front Psych. (2023) 14:1123445. doi: 10.3389/fpsyt.2023.1123445, 37265551 PMC10230301

[64] Song Y Yang F Sznajder K Yang X. Sleep quality as a mediator in the relationship between perceived stress and job burnout among Chinese nurses: a structural equation modeling analysis. Front Psych. (2020) 11:566196. doi: 10.3389/fpsyt.2020.566196, 33281640 PMC7691233

[65] Chang W Chang Y. Relationship between job satisfaction and sleep quality of female shift-working nurses: using shift type as moderator variable. Ind Health. (2019) 57:732–40. doi: 10.2486/indhealth.2018-0258, 30930373 PMC6885599

[66] Kader M Selander J Andersson T Albin M Bodin T Härmä M . Night and shift work characteristics and incident ischemic heart disease and atrial fibrillation among healthcare employees—a prospective cohort study. Scand J Work Environ Health. (2022) 48:520–9. doi: 10.5271/sjweh.4045, 35723926 PMC10539110

[67] Jang S Lee MK. Effects of anxiety focused nursing interventions on anxiety, cognitive function and delirium in neurocritical patients: a non-randomized controlled design. Nurs Crit Care. (2025) 30:e70062. doi: 10.1111/nicc.70062, 40396467 PMC12093422

[68] Killgore WDS Grandner MA Tubbs AS Fernandez F Doty TJ Capaldi Ii VF . Sleep loss suicidal ideation: the role of trait extraversion. Front Behav Neurosci. (2022) 16:886836. doi: 10.3389/fnbeh.2022.886836 36338878 PMC9630630

[69] Jiao W Lin J Deng Y Ji Y Liang C Wei S . The immunological perspective of major depressive disorder: unveiling the interactions between central and peripheral immune mechanisms. J Neuroinflammation. (2025) 22:10. doi: 10.1186/s12974-024-03312-3, 39828676 PMC11743025

[70] Zhou B Ma R Wang M Wang Y. Dose–response relationship between nighttime sleep duration and intrinsic capacity declines among Chinese elderly: a cross-sectional study from CHARLS. BMC Public Health. (2025) 25:1034. doi: 10.1186/s12889-025-22294-2, 40098027 PMC11917026

[71] Wang W Zhu Y Yu H Wu C Li T Ji C . The impact of sleep quality on emotion regulation difficulties in adolescents: a chained mediation model involving daytime dysfunction, social exclusion, and self-control. BMC Public Health. (2024) 24:1862. doi: 10.1186/s12889-024-19400-1, 38992632 PMC11241850

[72] Al-Hrinat J Al-Ansi AM Hendi A Adwan G Hazaimeh M. The impact of night shift stress and sleep disturbance on nurses quality of life: case in Palestine red crescent and Al-Ahli hospital. BMC Nurs. (2024) 23:24. doi: 10.1186/s12912-023-01673-3, 38185660 PMC10773077

[73] He H Wang J. The roles of loneliness and self-control in the association between social avoidance and nomophobia among college students. Sci Rep. (2025) 15:26782. doi: 10.1038/s41598-025-10789-2, 40702087 PMC12287310

[74] Liu X Liu T Zhou Z Wan F. The effect of fear of missing out on mental health: differences in different solitude behaviors. BMC Psychol. (2023) 11:141. doi: 10.1186/s40359-023-01184-5, 37127680 PMC10150542

[75] Jaworska D. Many faces of FOMO: a qualitative in-depth investigation of context-specific experiences, emotions, and coping strategies. PLoS One. (2025) 20:e330978. doi: 10.1371/journal.pone.0330978, 40892849 PMC12404441

[76] Qutishat M. Relationship between second victim syndrome and fear of missing out among nurses in Oman: implications for nursing practice. Nurs Crit Care. (2025) 30:e70011. doi: 10.1111/nicc.70011, 40040327

