# Supplementary material for: The impact of night shift workload on nurses’ depressive symptoms: a chain mediation analysis of sleep disturbances, social avoidance and fear of missing out
Source: Front Public Health. 2025 Dec 17;13:1723567. doi: 10.3389/fpubh.2025.1723567 (PMC12753973; doi:10.3389/fpubh.2025.1723567)
Supplement: Supplementary file 1 [file Table_1.docx]

**Table 1. Shapiro-Wilk normality test results**

| Variable | *W* | *P* |
| --- | --- | --- |
| Night shift workload | 0.906 | ＜0.001 |
| Sleep disturbances | 0.889 | ＜0.001 |
| Social avoidance and distress | 0.911 | ＜0.001 |
| Fear of missing out | 0.899 | ＜0.001 |
| Depressive symptoms | 0.965 | ＜0.001 |
